# Program Evaluation in Medical Education: An Overview of the Utilization-focused Approach

**DOI:** 10.3352/jeehp.2010.7.1

**Published:** 2010-06-15

**Authors:** Matt Vassar, Denna L. Wheeler, Machelle Davison, Johnathan Franklin

**Affiliations:** Oklahoma State University, Center for Health Sciences, Tulsa, OK, USA.

**Keywords:** Program Evaluation, Medical Education, Utilization-Focused Evaluation

## Abstract

Medical school administrators, educators, and other key personnel must often make difficult choices regarding the creation, retention, modification, or termination of the various programs that take place at their institutions. Program evaluation is a data-driven strategy to aide decision-makers in determining the most appropriate outcome for programs within their purview. The purpose of this brief article is to describe one program evaluation model, the utilization-focused approach. In particular, we address the focus of this model, the personal factor, the role of the evaluator, and the evaluation process. Based on the flexibility of this model as well as its focus on stakeholder involvement, we encourage readers to consider the utilization-focused approach when evaluating programs.

Medical education is a dynamic, rapidly-evolving enterprise. Technological advances such as simulation, virtual reality, and personal digital assistants, to name a few, have considerably altered the way in which medical students are educated and trained. By incorporating such technologies into the learning experience, administrators at various levels of medical training are faced with an important series of questions: Do these technologies sufficiently enhance student learning, and are the costs associated with implementing these technologies justifiable?

Questions such as these extend well beyond technologically-driven learning experiences. Consider medical school administrators attempting to determine whether an alternative admissions program is effective or if a new standardized patient approach might increase pass rates on high stakes exams. In the post-graduate years, residency directors need to know if trainees meet educational goals as well as ensure that their training programs are of sufficient quality. In each of these scenarios, the effectiveness of the program is questioned. The means for answering such questions are accomplished through a systematic and comprehensive program evaluation. Unfortunately, the majority of evaluations performed often lack the proper structure or theoretical framework to effectively guide the process.

According to Fitzpatrick, Sanders, and Worthen, program evaluation is defined as "the identification, clarification, and application of defensible criteria to determine an evaluation object's value (worth or merit) in relation to those criteria" [1]. In broad terms, this process involves determining standards to assess the quality, collecting appropriate information, and applying the standards to evaluate the effectiveness and value of the evaluation object.

There are many program evaluation models. A few examples include: objectives-oriented approaches, management-oriented approaches, participant-oriented approaches, expertise-oriented approaches, and logic models. These models have been adopted in various educational fields and have enjoyed widespread usage. Program evaluation models for medical education also exist but are much more limited. Durning, Hemmer, and Pangaro rightly note that research related to program evaluation in medical education is less developed than other educational fields [2]. Readers are referred to Gibson et al. [3], and Musick [4] for specific information related to evaluation models within the medical education domain.

Given the limited exposure of program evaluation models in medical education, the purpose of the current article is to briefly describe one model of program evaluation, the utilization-focused method. The authors feel that this model is well-suited for medical education, given its flexibility in approach as well as its focus on the active involvement of stakeholders. This active position promotes the stakeholders' investment in the process and increases the likelihood that they will see value in the results needed to affect change. In the next sections we will address the focus of this model, the personal factor, the role of the evaluator, and the evaluation process. For additional information regarding this evaluation model, readers are referred to Patton, the source from which the following information is based [5, 6].

Utilization-focused evaluation begins with the assumption that evaluations should be judged by their actual use and utility [5]. It is the responsibility of the evaluator to pay careful attention to the design of the evaluation, ensuring that each step taken from beginning to end affects intended use. The users (stakeholders) guide the evaluator and are responsible for deriving goals or objectives of the evaluation project.

Utilization-focused evaluation is context specific. It is not limited to a particular approach or methodology. Rather, it is a means of aiding users in determining the approach and methodology that works best within the context of their particular program. Situational responsiveness facilitates this interactive process between the evaluator and the users. With the expansive options available, users are able to choose the purpose of the evaluation (formative or summative), the data type (quantitative, qualitative or both), the type of research design (experimental, quasi-experimental, or non-experimental), and the evaluation focus (process, outcomes, costs, benefits, etc.). In addition, specific situational variables are always present and must be incorporated into the evaluation. Examples might include the diversity of the stakeholders, available resources, political factors, and interests. To briefly demonstrate a utilization-approach in contrast with other evaluation models, consider the example of stakeholders interested in evaluating a standardized patient program. An objectives-oriented method would establish clearly defined objectives that the program must meet in order to be effective, such as a pass rate percentage on a high stakes exam. In contrast, a participant-oriented evaluator might conduct qualitative interviews or focus groups of medical students or standardized patients to understand their perspectives in greater depth. Since utilization-focused evaluations are flexible and vary by context, they are able to incorporate elements from any program evaluation model to best meet the stakeholders' needs.

Utilization-focused evaluation is also very personal. In fact, the personal factor is highly emphasized. Given that there may be an unlimited number of stakeholders, each with competing interests, it is the responsibility of the evaluator to scale down potential stakeholders to a group of interested individuals who actively participate and care about the evaluation and the findings it generates. The evaluation seeks to serve their interests primarily. The evaluator often develops a strong working relationship with users and actively engages them throughout the evaluative process.

Within the context of evaluation, the role of the evaluator is often a key element. For example, if the goal of the evaluation is to provide generalizable knowledge of cause-effect relationships between the program and a particular outcome variable, the evaluator serves as a methodologist with expertise in research design. Consider a second example in which the purpose of an evaluation is to examine a program's overall merit. In this case, the evaluator must become a judge. The primary role of the utilization-focused evaluator is that of negotiator. In this function, the evaluator negotiates with users the roles in which he or she will take part. Patton also expresses the role of the evaluator as active-reactive-adaptive. Utilization-focused evaluators are, first of all, active in deliberately and calculatedly identifying intended users and focusing useful questions. They are reactive in listening to intended users and responding to what they learn about the particular situation in which the evaluation unfolds. They are adaptive in altering evaluation questions and designs in light of their increased understanding of the situation and changing conditions [5].

As with any program evaluation, utilization-focused evaluations are systematic and follow a logical sequence. First, the primary users/stakeholders of the evaluation are identified. Next, the evaluator and users commit to the purpose of the evaluation and derive a focus to address particular goals or to take into account the program's theory of action. The third step of the process involves the selection of the research design and measurement considerations. Sampling issues, the nature of the data, and the quality of assessment devices are but a few of the decisions that must be made during this phase. Following data collection, users are brought together to assist in interpreting findings and making judgments based on the data to derive recommendations. In the final step, decisions are made about the dissemination of the evaluation report.

Medical schools are required to continuously assess program effectiveness, especially in light of recent budget cuts and diminishing resources. Given that programs are under increased scrutiny, effective program evaluation has never been more vital. For these reasons, the previous description provides a glance into a program evaluation model that is useful in a wide array of contexts within medical education. One of its key benefits is that it is a flexible approach that can be useful in answering a wide variety of programmatic questions. It also actively includes principal stakeholders in the decision making process, increasing the likelihood that results will be used to guide program development and decision making. For these reasons, we encourage those interested in evaluating a particular program to consider the utilization-focused approach.
